# Effect of *Lactiplantibacillus plantarum* DSW3805 Isolated from Kimchi for Gut Health Attenuating Colonic Inflammation in a Dextran Sulfate Sodium-Induced Mouse Model

**DOI:** 10.3390/nu17071259

**Published:** 2025-04-03

**Authors:** Na-Kyoung Lee, Yunjung Lee, Da-Soul Shin, Yong-Min Choi, Jinhyeuk Lee, Eunju Park, Hyun-Dong Paik

**Affiliations:** 1 Department of Food Science and Biotechnology of Animal Resources, Konkuk University, Seoul 05029, Republic of Korea; lnk11@konkuk.ac.kr; 2 Department of Food and Nutrition, Kyungnam University, Changwon 51767, Republic of Korea; hjlee@kyungnam.ac.kr (Y.L.); dasoul920@naver.com (D.-S.S.); 3 Daesang Wellife, Seoul 03130, Republic of Korea; ymchoi@daesang.com (Y.-M.C.); jinlee@daesang.com (J.L.)

**Keywords:** gut health, probiotics, immunity, inflammatory, gut microbiota

## Abstract

**Background/Objectives:** *Lactiplantibacillus plantarum* DSW3805 was isolated from Korean kimchi samples to examine its effect in a dextran sulfate sodium (DSS)-induced mouse model. **Methods:** To induce colitis, mice were treated with DSS for one week before sacrifice (*n* = 8 per group, total *n* = 40). *Lacticaseibacillus rhamnosus* GG (10^9^ CFU/day) or probiotics (*L. plantarum* DSW3805; 10^8^ or 10^9^ CFU/day) were administered for two weeks. To assess colitis damage, we evaluated the disease activity index, colon tissue, inflammatory factors, the microbiome, short-chain fatty acids, and intestine-related factors. **Results:** DSS induced colonic tissue damage (colon length, mucus thickness, and colonic crypts), and *L. plantarum* DSW3805 alleviated the tissue damage. Induced inflammation was reduced by inhibiting TNF-α, IFN-γ, IL-1β, IL-6, IgA, IgG, LTB4, PGE2, and NF-κB protein expression. The ratio of Firmicutes to Bacteroidetes in the PC group (DSS-treated control) was lower than that in the NC (DSS-nontreated control); *L. plantarum* DSW3805 increased the ratio. Higher concentrations of acetic, propionic, and butyric acids were detected in probiotic groups. In addition, harmful factors, such as calprotectin and β-glucuronidase, were reduced in the probiotic groups. **Conclusions:** *L. plantarum* DSW3805 alleviates gut damage by colitis; therefore, it can be used as a functional food to improve gut health.

## 1. Introduction

Gut dysbiosis is influenced by bacterial infections, inflammatory bowel disease such as ulcerative colitis and Crohn’s disease, and general digestive difficulties (diarrhea, constipation, and gas). Especially, gut health is a temporary or chronic disorder that causes a variety of gastrointestinal (GI) symptoms [1]. GI disorders is a term used to describe a set of symptoms caused by several etiologies, including the risk of infection, malabsorption due to infectious diseases, small intestinal bacterial overgrowth, antibiotic use, altered gut microbiome, and an excessive exercise response leading to diarrhea or constipation-like symptoms. Illnesses related to various factors that cause psychological stress, such as depression [2]. The goal of gut health is to relieve symptoms, such as cramps, pain, and diarrhea, so that the patient’s symptoms improve. This helps improve the quality of life by improving constipation and abdominal bloating [3].

Probiotics improve the bowel movement, intestinal transit time, and stool consistency [4]. The anti-inflammatory properties of lactobacilli and bifidobacteria have been exploited to alleviate gut-associated inflammation in a colitis model [5]. The probiotic mixture, VSL#3 or *Lacticaseibacillus rhamnosus* GG (LGG), is well-known for its beneficial effects in preventing GI infections or diarrhea by stimulating the immune response [6]. Probiotics influenced intestinal conditions by SCFAs (short-chain fatty acids including acetate, butyrate, and propionate) production, reduction in inflammatory cytokines (IL-1α, IL-1β, IL-6, IL-12, TNF-α, and IFN-γ), and the gut barrier integrity [7]. These results suggest that intestinal homeostasis can be maintained and intestinal inflammation can be alleviated.

For gut health, 5-hydroxytryptamine or serotonin type 3 (5-HT3) receptor agonists are used to reduce pain sensation, and loperamide, eluxadoline, and probiotics are used as antimotility agents [8,9]. However, reported adverse effects of loperamide and eluxadoline include constipation [10]. In addition, treatments for inflammatory bowel disease (IBD) have been used as anti-inflammatory medicines (mesalamine, balsalazide, and olsalazine) and immunomodulators (azathioprine, mercaptopurine, and methotrezte). Therefore, probiotics, synbiotics, and natural plant extracts, such as pomegranate peel extract, have been used as alternatives [11,12].

Many probiotics are known to promote intestinal health by enhancing epithelial function, modulating microbial homeostasis of intestinal flora or serotonin production via the gut–brain axis [13,14,15]. Dextran sulfate sodium (DSS)-induced colitis in animal models has been used to investigate human colitis. This study aimed to investigate the alleviation of intestinal conditions in a DSS-induced colitis model using new probiotics isolated from kimchi.

## 2. Materials and Methods

### 2.1. Preparations of LAB Samples

*Lactiplantibacillus plantarum* DSW3805 was isolated from kimchi in Korea. *L. rhamnosus* GG, supplied by the Korean Collection for Type Cultures (Daejeon, Republic of Korea), was used as a control. These strains were cultured as probiotics in the MRS medium. The cultured strain was centrifuged and resuspended in PBS (HyClone, Logan, UT, USA). The harvested strain was added to skim milk, lyophilized, and used to develop animal models.

### 2.2. Animal Groups and Experimental Design

**Figure 1:**
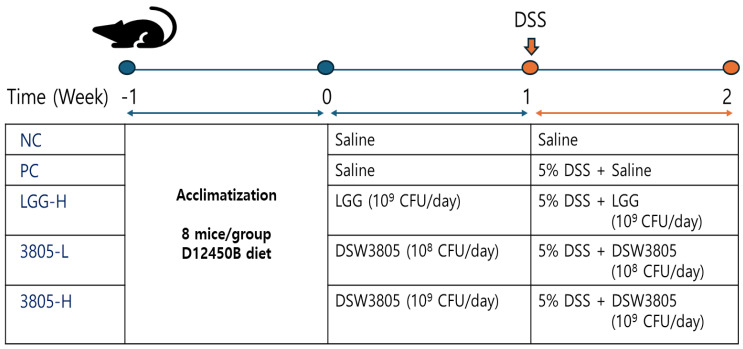
Animal study for a DSS-induced colitis model. DSS, dextran sulfate sodium; LGG, *L. rhamnosus* GG; DSW3805, *L. plantarum* DSW3805.

A total of 40 male ICR mice (weight, 28.4 ± 2.2 g; age, 6 weeks old) were purchased from Koatech (Pyeongtaek, Republic of Korea) and induced with colitis using DSS (Figure 1) [16,17]. The mice were housed (3–4 per cage) at 23 ± 1˚C and 53 ± 2% relative humidity with a 12 h light/dark cycle, and the cages were lined with sawdust. After one week of acclimatization, the mice were randomly assigned to five groups (*n* = 8) based on their body weight, as follows: (1) NC (nontreated control), (2) PC (5% DSS treated control), (3) LGG-H (5% DSS with 10^9^ CFU/day of LGG), (4) 3805-L (5% DSS with 10^8^ CFU/day of DSW3805), and (5) 3805-H (5% DSS with 10^9^ CFU/day of DSW3805), with 4 replicates per treatment and two mice per cage (200 × 260 × 130 mm, polycarbonate). All the groups were fed a 10% kcal fat diet [D12450B (Research Diets, Inc., Yongin, Republic of Korea)]. Probiotics were pre-administered to the mice for 1 week using a zonde. The NC and PC groups were administered with equal volumes of PBS. Next, 5% DSS was added to the water to induce colitis for 1 week. The probiotics were administered until the end of the experiment. All experimental protocols were performed in accordance with the guidelines of the Institutional Animal Care Committee of Kyungnam University (approval code: KUICA-24-04; approval date: 5 August 2024). This study is reported in accordance with ARRIVE guidelines for animal studies.

### 2.3. Clinical Evaluation

The disease activity index (DAI) was analyzed to evaluate the degree of colitis induction during DSS administration. Weight loss, diarrhea, hematochezia, and rectal bleeding were visually checked and evaluated according to Table S1, calculated on a scale of 0–4 points, and then summed [18]. No other adverse reactions were observed, and all mice were sacrificed after 2 weeks of treatment under anesthesia induced by isoflurane (4 mL/kg). Body and spleen weights were measured, and the length of the large intestine was measured using a ruler.

### 2.4. Colon Tissue Observation Using Eye and Microscope

The degree of colonic lesions, such as redness, thickening of the mucosal tissue, and ulcers due to tissue necrosis, was visually observed according to the intestinal histological evaluation scale.

The distal colon was fixed in formalin and prepared as paraffin-embedded tissue specimens. The tissues were sectioned at 4-µm thickness and then stained with hematoxylin and eosin. To analyze the degree of proliferation of the colonic mucosa, the thickness of the mucosa was measured vertically at 100× magnification using the Motic Digital Slide Assistant Scanner program (Motic^®^, Vancouver, BC, Canada).

### 2.5. Quantification of Cytokine, Immunoglobulin, and Inflammatory Factors Using Enzyme-Linked Immunosorbent Assays

The colon was homogenized in ice-cold PBS, followed by centrifugation for 15 min at 1500× *g* and 4 °C. The concentration of cytokines (IL-6, TNF-α, IFN-γ, and IL-1β), immunoglobulin (IgG and IgA), and inflammatory factors (LTB4, PGE2, and NF-κB) were determined in colon tissue supernatant using an ELISA kit (MyBioSource, San Diego, CA, USA).

### 2.6. Microbiome Analysis, Beneficial, and Harmful Bacteria

Microbiome analyses were performed at the Korean Research Institute of Biomedical Science (Daejeon, Republic of Korea). DNA was extracted from feces using a QIAamp PowerFecal Pro DNA kit (Qiagen, Hilden, Germany). PCR was performed according to Illumina 16S V3-V4 amplicon sequencing (Illumina, San Diego, CA, USA) and identified using the Illumina MiSeq system (Illumina).

To analyze beneficial and harmful bacteria, a cell counting assay was performed. Feces (0.1 g) were inoculated into 900 μL of brain heart infusion broth containing 0.05% l-cysteine hydrochloride hydrate. Dilutions were spread on selective agar plates and incubated at 37 °C to count the number of colonies. Lactobacilli MRS, *Bifidobacterium*-selective agar, mannitol salt agar, and McConkey agar were used for *Lactobacillus*, *Bifidobacterium*, *Staphylococcus aureus*, and *Escherichia coli*, respectively.

### 2.7. Analysis of Short Chain Fatty Acid, Calprotectin, and β-Glucuronidase

Stool samples were added to an aqueous solution [1 N HCl (0.25 mL) and 0.1 M isobutanol (0.025 mL)] and vortexed for 10 min. The samples were extracted with diethylether. The supernatant was collected after centrifugation at 12,000× *g* for 5 min. One microliter of the supernatant was injected into a gas chromatograph-MASS (Hewlett-Packard Model 7890, Palo Alto, CA, USA) equipped with a DB-FATWAX Ultra Inert column for SCFAs analysis.

The monoclonal antibody against calprotectin was covalently bound to the solid phase with calprotectin in the sample to form an antigen–antibody complex, after which the unbound components were removed through a washing process. The enzyme then underwent a secondary reaction with an antibody against labeled calprotectin to form a calprotectin–conjugate complex. Unbound components were removed by washing. The reaction was stopped, and the degree of fluorescence was measured for calprotectin calculations.

To analyze β-glucuronidase activity, 100 μL of enzyme solution, 380 μL of 0.1 M potassium phosphate buffer, and 20 μL of 10 mM p-nitrophenyl-β-glucuronide as a substrate were added and reacted at 37 °C for 60 min. The reaction was stopped by adding 500 μL of 0.5 N NaOH, and centrifugation was performed at 3000 rpm. The supernatant was collected, and its absorbance was measured at 405 nm.

### 2.8. Data Analysis

All data were represented as the mean ± standard error of three replicates. The significance of the differences between the control and treatment groups was evaluated using one-way ANOVA at a 95% confidence level. Statistical significance was set at *p* < 0.05. All calculations were performed using SPSS for Windows, version 20.0 (SPSS Inc., Chicago, IL, USA).

## 3. Results

### 3.1. Clinical Evaluation and Intestinal Damage in DSS-Induced Mouse Models

To induce colitis, 5% DSS was supplied to the experimental animals, excluding the control group (NC), and disease activity was measured to confirm the degree of colitis induction after seven days.

**Figure 2:**
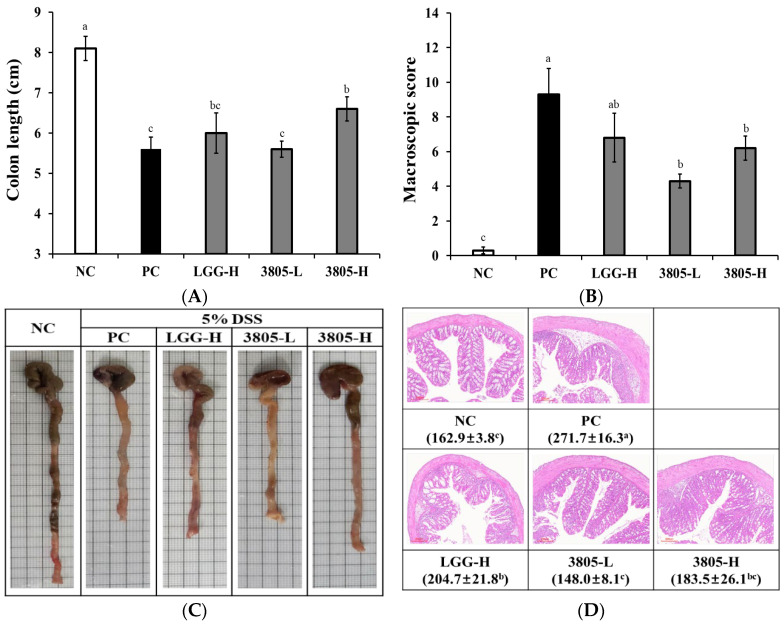
Effects of *Lactiplantibacillus plantarum* DSW3805 on clinical and histological symptoms in DSS-induced colitis models (*n* = 8). (**A**) Colon length; (**B**) macroscopic score, (**C**) images of the colon, and (**D**) H&E stained colonic section and mucosal thickness. NC, DSS-nontreated model; PC, DSS-treated control; LGG-H, DSS with 10^9^ CFU/day of LGG; 3805-L, DSS with 10^8^ CFU/day of *L. plantarum* DSW3805; 3805-H, DSS with 10^9^ CFU/day of *L. plantarum* DSW3805. Data are presented as mean ± standard error of triplicate experiments. ^a–c^ Different letters on the error bars represent significant differences (*p* < 0.05). Macroscopic score was followed by “Visual evaluation criteria” [19]. LGG, *L. rhamnosus* GG.

At the time of animal sacrifice, the body weight was 33.6 g, 29.9 g, 32.2 g, 30.5 g, and 31.0 g in NC, PC, LGG-H, 3805-L, and 3805-H groups, respectively; colon length was 8.1 cm, 5.6 cm, 6.0 cm, 5.8 cm, and 6.6 cm, respectively; spleen weight was 0.08 g, 0.15 g, 0.12 g, 0.11 g, and 0.13 g, respectively (Table 1 and Figure 2). Compared with the PC (DSS group), the colon length of the probiotics group tended to increase, and a significant difference was observed in the 3805-H group (*p* < 0.05).

In the PC group, the DAI score through visual observation was significantly increased compared to the NC (Table 1, *p* < 0.05). There was no significant difference in the DAI scores between the 3805-L and 3805-H groups according to intake concentration. However, the DAI score of the 3805-H group significantly decreased compared to the PC group (*p* < 0.05). In particular, the diarrhea score in the PC group (3.33) was increased by DSS treatment compared with that in the NC (0). Meanwhile, 3805-L (3.05) and 3805-H (2.89) groups showed lower diarrhea scores than the LGG-H group (3.22) (*p* < 0.05).

Histopathological analysis results showed that colon tissues of the NC had normal crypts in the mucosa, submucosa, and muscularis mucosa (Figure 2D). Compared with the NC, the PC significantly showed typical inflammatory changes in the colonic tissue, such as loss of colonic crypts, thickening of the colonic mucosa, and infiltration of inflammatory cells (*p* < 0.05). However, the administration of probiotics offset the tissue characteristics of DSS-induced colitis and showed changes similar to those of the NC. It showed a normal colonic tissue morphology. The mucosal thickness of the PC group (271.7 μm) showed significant changes compared with that of NC (162.9 μm). The 3805-L (148.0 μm) and 3805-H (183.5 μm) groups showed similar mucosal thickness to NC compared with that of the LGG-H group (204.7 μm) (*p* < 0.05). Macroscopic scores were significantly different between the NC (3.0) and PC (34.3) groups (*p* < 0.05). In addition, LGG (15.0), 3805-L (23.0), and 3805-H (19.7) groups showed less damage than the PC (6.2) group (*p* < 0.05).

### 3.2. Immune Regulation Effect in Intestinal Tissue

All the probiotic-treated groups showed significant reduction in proinflammatory cytokines, TNF-α, IFN-γ, IL-1β, and IL-6, in intestinal tissue compared to the PC group in Figure 2 (*p* < 0.05). The production of all the tested cytokines in the PC group was significantly higher than that in the NC group. In the 3805 groups, the production of TNF-α, IFN-γ, IL-1β, and IL-6 was significantly reduced compared to the PC group, and there was no difference in intake concentration.

**Figure 3:**
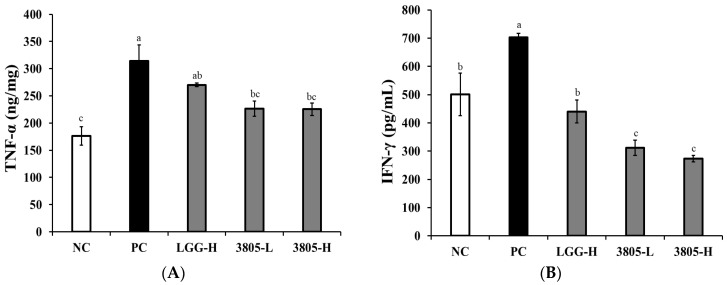

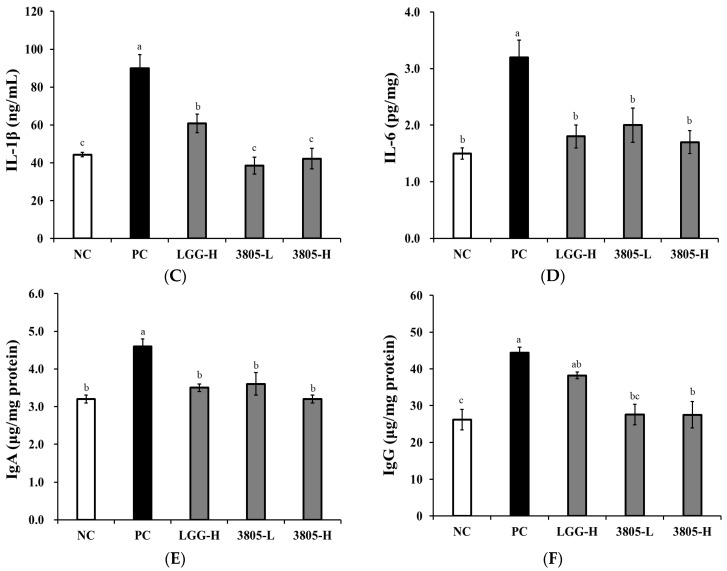
Effects of *Lactiplantibacillus plantarum* DSW3805 on immune regulation in DSS-induced colitis models (*n* = 8). (**A**) TNF-α, (**B**) IFN-γ, (**C**) IL-1β, (**D**) IL-6, (**E**) IgA, and (**F**) IgG. NC, DSS-nontreated model; PC, DSS-treated control; LGG-H, DSS with 10^9^ CFU/day of LGG; 3805-L, DSS with 10^8^ CFU/day of *L. plantarum* DSW3805; 3805-H, DSS with 10^9^ CFU/day of *L. plantarum* DSW3805. Data are presented as mean ± standard error of triplicate experiments. ^a–c^ Different letters on the error bars represent significant differences (*p* < 0.05). LGG, *L. rhamnosus* GG.

IgA and IgG concentrations in the intestinal tissue were measured using ELISA. The PC group showed significantly higher IgA and IgG concentrations than the NC (Figure 3, *p* < 0.05). The 3805 groups had significantly lower IgA concentrations than those of the PC group, and there was no difference in the intake concentration.

**Figure 4:**
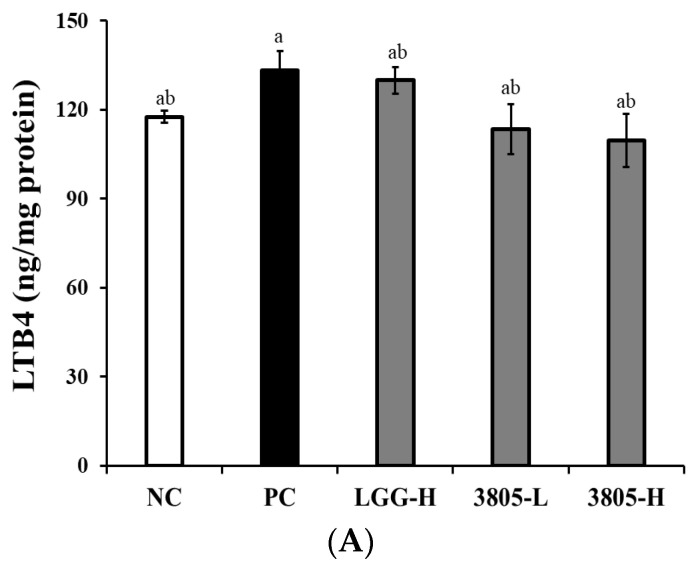

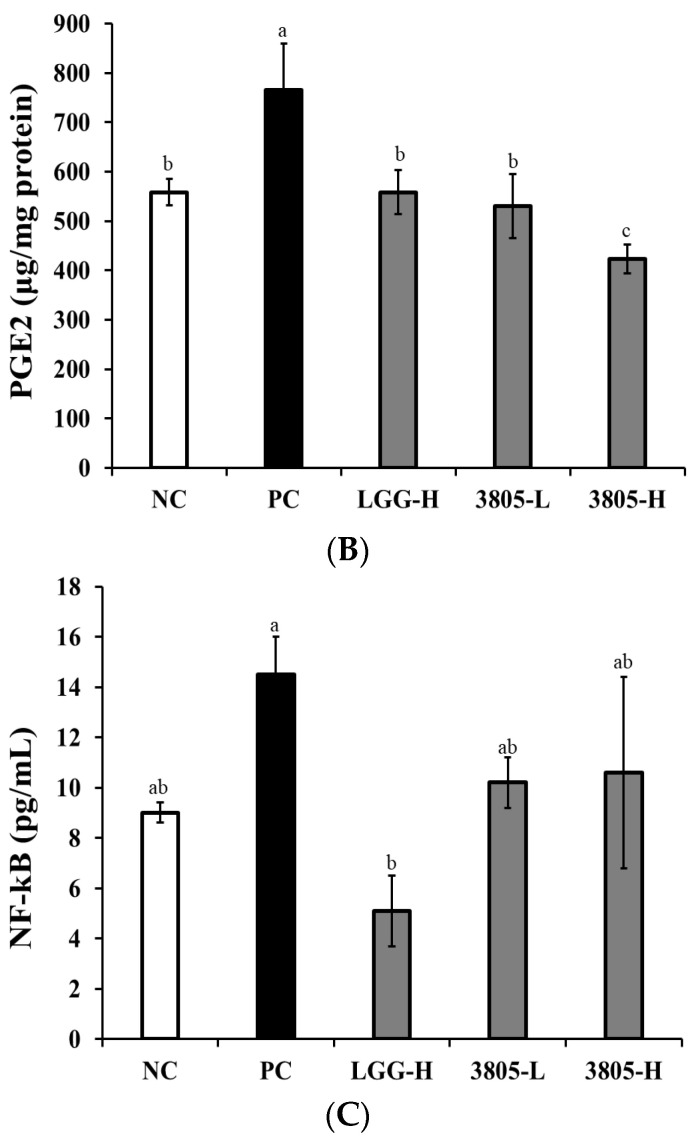
Effects of *Lactiplantibacillus plantarum* DSW3805 on inflammation-related factors in DSS-induced colitis models (*n* = 8). (**A**) LTB4, (**B**) PGE2, and (**C**) NF-κB. NC, DSS-nontreated model; PC, DSS-treated control; LGG-H, DSS with 10^9^ CFU/day of LGG; 3805-L, DSS with 10^8^ CFU/day of *L. plantarum* DSW3805; 3805-H, DSS with 10^9^ CFU/day of *L. plantarum* DSW3805. Data are presented as mean ± standard error of triplicate experiments. ^a–c^ Different letters on the error bars represent significant differences (*p* < 0.05). LGG, *L. rhamnosus* GG.

The PC group showed increased production of LTB4, PGE2, and NF-kB compared to the NC, but the difference was not statistically significant (Figure 4). In 3805 groups, LTB4, PGE2, and NF-kB production decreased compared with those in the PC group, and there was no difference in intake concentration.

### 3.3. Microbiome Analysis and Beneficial and Harmful Bacteria in Feces

**Figure 5:**
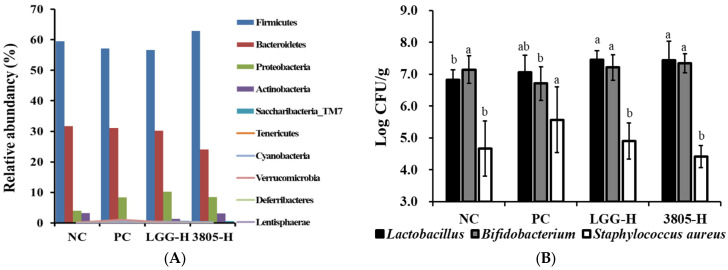

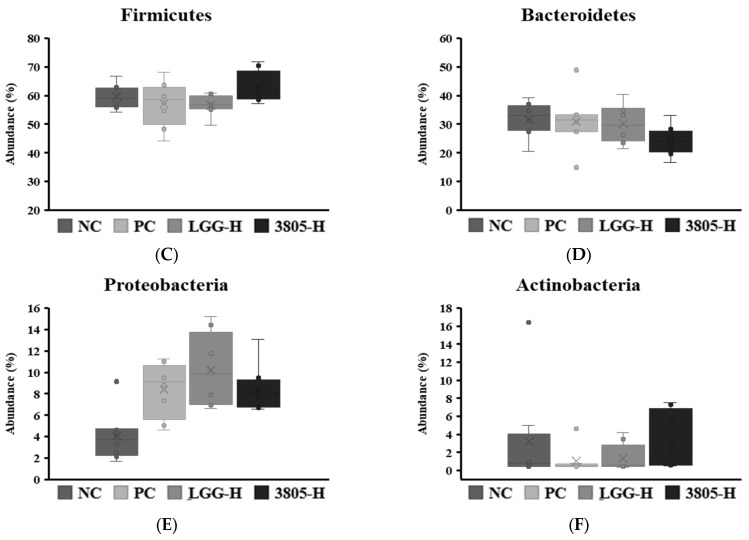
Effects of *Lactiplantibacillus plantarum* DSW3805 on (**A**) relative microbial abundance, (**B**) beneficial and harmful microorganisms, (**C**) Firmicutes abundance, (**D**) Bacteriodetes abundance, (**E**) Proteobacteria abundance, and (**F**) Actinobacteria abundance in DSS-induced colitis models (*n* = 8). PC, DSS-treated control; LGG-H, DSS with 10^9^ CFU/day of LGG; 3805-L, DSS with 10^8^ CFU/day of *L. plantarum* DSW3805; 3805-H, DSS with 10^9^ CFU/day of *L. plantarum* DSW3805. LGG, *L. rhamnosus* GG. ^a,b^ Different letters on the error bars represent significant differences (*p* < 0.05).

The ratio of Firmicutes to Bacteroidetes was determined using microbiome analysis. The ratio was 1.88:1, 1.84:1, 1.88:1, and 2.61:1 in the NC, PC, LGG-H, and 3805-H groups, respectively. Firmicutes were increased and Bacteroidetes bacteria were reduced in the 3805 groups compared with those in the PC group (Figure 5).

The beneficial and harmful bacteria in the fecal samples were spread on each selective medium, and the results of the culture are shown in Figure 5B. The number of *Lactobacillus* strains was 6.82, 7.05, 7.46, and 7.18 log CFU/g in the NC, PC, LGG-H, and 3805-H groups, respectively; however, the results were not significant. *Bifidobacterium* strains were confirmed to be 7.14, 6.71, 7.28, and 7.16 log CFU/g in the NC, PC, LGG-H, and 3805-H groups, respectively.

*S. aureus* abundance was confirmed to be 4.67, 5.57, 4.90, and 4.41 log CFU/g in NC, PC, LGG-H, and 3805-H groups, respectively. Among the samples, the 3805-H group had the least amount of *S. aureus*. *E. coli* was either not detected or had low values.

### 3.4. SCFAs, Calprotectin, and β-Glucuronidase Analysis in Feces

**Figure 6:**
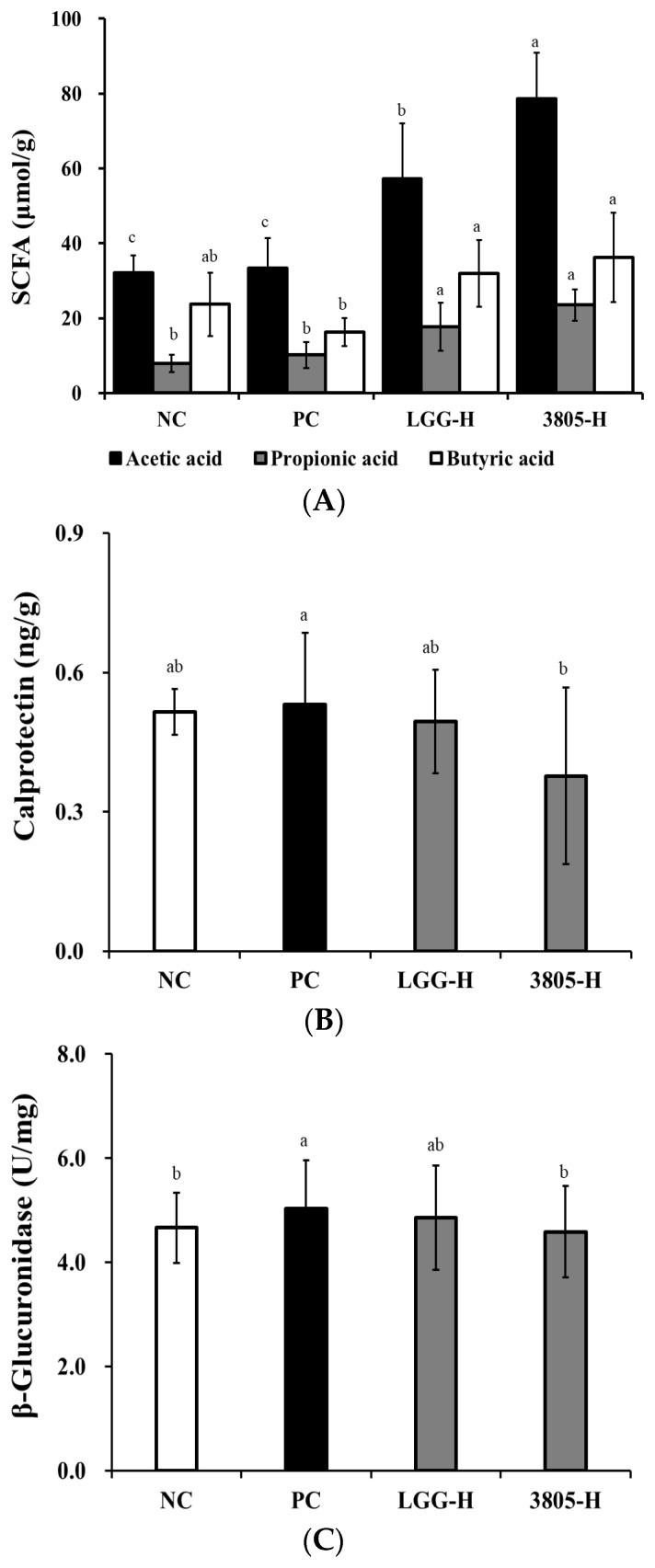
Effects of *Lactiplantibacillus plantarum* DSW3805 on (**A**) short chain fatty acid, (**B**) calprotectin, and (**C**) β-glucuronidase in DSS-induced colitis models. PC, DSS-treated control; LGG-H, DSS with 10^9^ CFU/day of LGG; 3805-L, DSS with 10^8^ CFU/day of *L. plantarum* DSW3805; 3805-H, DSS with 10^9^ CFU/day of *L. plantarum* DSW3805. Data are presented as mean ± standard error of triplicate experiments. ^a–c^ Different letters on the error bars represent significant differences (*p* < 0.05). LGG, *L. rhamnosus* GG.

The SCFAs content of the feces was determined using the GC mass. Acetic, propionic, and butyric acids were detected in all samples (Figure 6A, *p* < 0.05). SCFAs production in the NC and PC groups did not differ significantly. The 3805-H group showed a higher total SCFAs production than that in the PC group. Calprotectin has been identified as a factor in inflammatory bowel disease and is known to increase during inflammation. As a result of testing calprotectin in feces, it was confirmed that it decreased in the 3805-H (0.38 ng/g) group compared with that in the PC (0.53 ng/g) (Figure 6B, *p* < 0.05). Additionally, the activity of β-glucuronidase, a harmful enzyme, was higher in the PC group (5.03), while the 3805-H (4.58 U/mg) group had lower activity than that of the LGG-H group (4.85 U/mg) (Figure 6C, *p* < 0.05).

## 4. Discussion

DSS causes epithelial cell injury and leads to immune responses that alter the mucosal barrier function throughout the colonic epithelium and has been extensively used to induce colitis in mice [20,21,22,23]. The pathogenesis of colitis caused by DSS is unclear, but can be summarized as the following mechanisms: (1) reduction in resistance and death of mucosal epithelial cells due to increased osmosis of the colonic mucosa; (2) intestinal-specific T cell induction of immune inflammatory response; (3) increased expression of cytokines, such as TNF-α, IFN-γ, and IL-10; and (4) changes in the intestinal bacterial environment. Administration of DSS induces a highly reproducible acute inflammation confined to the colon, which is characterized by erosions/ulcers, loss of crypts, and infiltration of granulocytes.

In this study, we investigated a DSS-induced colitis model and found that *L. plantarum* 3805 alleviated DSS-induced diarrhea and colitis by reducing proinflammatory cytokines and modulating gut microbiota.

The histopathological analysis of the colon and DAI scores, including the diarrhea score, showed improvements in colonic histology in all probiotic-treated mice (Figure 2 and Table 1). Inflammatory infiltrates were often observed in both the small and large intestines in some patients with intestinal problems [24]. However, *L. plantarum* 3805 treatment resulted in the alleviation of inflammatory infiltrates and a longer colon length than with LGG treatment.

Inflammatory cytokines are associated with gut inflammation and gut motility in the mucus layer. TNF-α, IFN-γ, IL-10, and IL-1β have been reported on the mucous layer or via osmosis [25,26,27]. The anti-inflammatory effect of probiotics has been confirmed TNF-a, IFN-γ, IL-10, and IL-1β in the DSS-induced colitis mouse model. To identify the mechanism of colitis alleviation, LTB4, PGE2, and NF-κB production was confirmed to be lower in the 3805 groups than in the PC group (Figure 3 and Figure 4). Colitis-related factors can result in diarrhea, abdominal pain, or bleeding [28]. In addition, *L. plantarum* 3805 influenced the expression of ZO-1 and occludin, which are related to gut motility in intestinal cells.

Gut health is closely related to the gut microbiota and decreases the ratio of Firmicutes to Bacteroidetes (F:B) in colitis [29]. In our study, Figure 5 shows a reduction in harmful microbiota, and colon inflammation was alleviated by an increase in the F:B ratio. *L. reuteri* DSM 17938 and *L. plantarum* AN1 increase the F:B ratio [30,31]. *Bifidobacterium* is substantially associated with gut health [32,33]. In our study, the PC group decreased *Bifidobacterium* and *Actinobacteria*, including *Bifidobacterium* in fecal samples, whereas the NC and probiotic-treated groups increased. In particular, *S. aureus* and *Proteobacteria,* including *Campylobacter* or *Helicobacter*, were found to decrease in the 3805 group compared to the PC group.

SCFAs are the end products of anaerobic colonic bacterial fermentation and influence intestinal motility [34]. Figure 6A shows that the change in SCFAs in feces between the NC and PC groups was not significant; however, propionic acid increased in the PC group compared with that in the NC group. Butyric acid showed the greatest increase in the 3805 group. Butyric acid plays a major role in maintaining gut barrier integrity and reducing intestinal inflammation [35]. Fecal calprotectin, a biomarker of intestinal inflammation, represents the severity of gut health [36,37]. In our study, the PC (0.53 ng/g) and NC (0.51 ng/g) groups did not have significant differences in calprotectin (Figure 6B). In addition, LGG (0.50 ng/g) and 3805 (0.38 ng/g) groups reduced the calprotectin levels. β-glucuronidase can convert pre-carcinogens into carcinogens; therefore, it is a harmful fecal enzyme [38]. β-Glucuronidase in the PC group was increased, while the 3805 group was reduced, similar to NC (Figure 6C). Therefore, clinical research is needed to confirm the preventive potential of *L. plantarum* DSW3805.

## 5. Conclusions

In this study, we investigated the alleviating effects of the newly isolated *L. plantarum* DSW3805 in a DSS-induced colitis model. *L. plantarum* DSW3805 helps maintain gastric morphology and immune status by modulating cytokines and inflammatory factors. Based on fecal examinations, this strain could modulate intestinal microflora, SCFAs, calprotectin, and β-glucuronidase. Therefore, *L. plantarum* DSW3805 may help gut health.

## Figures and Tables

**Table 1:** Effect of *Lactobacillus* strains on the body weight, spleen weight, and disease activity index of 7 days in DSS-induced mice (*n* = 8).

	NC	PC	LGG-H	3805-L	3805-H
Body weight (g)	33.6 ± 1.3 ^a^	29.9 ± 0.9 ^b^	32.2 ± 0.8 ^ab^	30.5 ± 0.9 ^b^	31.0 ± 0.9 ^ab^
Spleen weight (g)	0.08 ± 0.01 ^c^	0.15 ± 0.01 ^a^	0.12 ± 0.01 ^ab^	0.11 ± 0.01 ^b^	0.13 ± 0.01 ^ab^
Disease activity index of 7 day (Score)	0.0 ± 0.0 ^c^	13.3 ± 0.6 ^a^	11.4 ± 0.7 ^b^	12.2 ± 0.7 ^ab^	10.9 ± 0.7 ^b^
Weight loss	0.00 ± 0.00 ^b^	3.00 ± 0.24 ^a^	2.67 ± 0.29 ^a^	3.20 ± 0.20 ^a^	2.89 ± 0.26 ^a^
Diarrhea	0.00 ± 0.00 ^b^	3.33 ± 0.17 ^a^	3.22 ± 0.15 ^a^	3.05 ± 0.23 ^a^	2.89 ± 0.18 ^a^
Hematochezia	0.00 ± 0.00 ^c^	3.22 ± 0.15 ^a^	3.11 ± 0.11 ^ab^	2.90 ± 0.10 ^b^	3.00 ± 0.00 ^ab^
Rectal bleeding	0.00 ± 0.00 ^d^	3.78 ± 0.15 ^a^	2.75 ± 0.25 ^c^	3.33 ± 0.17 ^ab^	3.17 ± 0.17 ^bc^

NC, DSS-nontreated model; PC, DSS-treated control; LGG-H, DSS with 10^9^ CFU/day of LGG; 3805-L, DSS with 10^8^ CFU/day of *L. plantarum* DSW3805; 3805-H, DSS with 10^9^ CFU/day of *L. plantarum* DSW3805. Data are presented as mean ± standard error of triplicate experiments. Different letters on the error bars represent significant differences (*p* < 0.05). LGG, *L. rhamnosus* GG.

## Data Availability

The data presented in this study are available on request.

## Supplementary Materials

The following supporting information can be downloaded at: https://www.mdpi.com/article/10.3390/nu17071259/s1, Table S1: Evaluation criteria of disease activity index.
