# Supplementary material for: Effect of Lactiplantibacillus plantarum DSW3805 Isolated from Kimchi for Gut Health Attenuating Colonic Inflammation in a Dextran Sulfate Sodium-Induced Mouse Model
Source: Nutrients. 2025 Apr 3;17(7):1259. doi: 10.3390/nu17071259 (PMC11990075; doi:10.3390/nu17071259)
Supplement: Supplementary file 1 [file nutrients-17-01259-s001.zip › nutrients-3509839-supplementary.pdf]

Supplementary Material

Table S1. Evaluation criteria of disease activity index [15]

Score	Weight loss (%)	Current of stools	Bloody stool	Anal bleeding
0	0	Normal	No bloody stool	No anal bleeding
1	1~5%	Soft stools	Red stool	No anal bleeding
2	6~10%	Loose stools	Bloody stool	No anal bleeding
3	10~20%	Diarrhea	Clear, red stool mixed with blood	Anal bleeding
4	>20%	Diarrhea	Red stool mixed with blood	Severe anal bleedin g
